# ATP-sulfurylase, sulfur-compounds, and plant stress tolerance

**DOI:** 10.3389/fpls.2015.00210

**Published:** 2015-04-07

**Authors:** Naser A. Anjum, Ritu Gill, Manjeri Kaushik, Mirza Hasanuzzaman, Eduarda Pereira, Iqbal Ahmad, Narendra Tuteja, Sarvajeet S. Gill

**Affiliations:** ^1^ Centre for Environmental and Marine Studies & Department of Chemistry, University of Aveiro, AveiroPortugal; ^2^ Stress Physiology and Molecular Biology Lab, Centre for Biotechnology, Maharshi Dayanand University, RohtakIndia; ^3^ Department of Agronomy, Faculty of Agriculture, Sher-e-Bangla Agricultural University, DhakaBangladesh; ^4^ Centre for Environmental and Marine Studies & Department of Biology, University of Aveiro, AveiroPortugal; ^5^ Plant Molecular Biology Group, International Centre for Genetic Engineering and Biotechnology, New DelhiIndia

**Keywords:** ATP-sulfurylase, sulfur assimilation, organic S-compounds, stress tolerance

## Abstract

Sulfur (S) stands fourth in the list of major plant nutrients after N, P, and K. Sulfate (SO_4_^2-^), a form of soil-S taken up by plant roots is metabolically inert. As the first committed step of S-assimilation, ATP-sulfurylase (ATP-S) catalyzes SO_4_^2-^-activation and yields activated high-energy compound adenosine-5^′^-phosphosulfate that is reduced to sulfide (S^2-^) and incorporated into cysteine (Cys). In turn, Cys acts as a precursor or donor of reduced S for a range of S-compounds such as methionine (Met), glutathione (GSH), homo-GSH (h-GSH), and phytochelatins (PCs). Among S-compounds, GSH, h-GSH, and PCs are known for their involvement in plant tolerance to varied abiotic stresses, Cys is a major component of GSH, h-GSH, and PCs; whereas, several key stress-metabolites such as ethylene, are controlled by Met through its first metabolite *S*-adenosylmethionine. With the major aim of briefly highlighting S-compound-mediated role of ATP-S in plant stress tolerance, this paper: (a) overviews ATP-S structure/chemistry and occurrence, (b) appraises recent literature available on ATP-S roles and regulations, and underlying mechanisms in plant abiotic and biotic stress tolerance, (c) summarizes ATP-S-intrinsic regulation by major S-compounds, and (d) highlights major open-questions in the present context. Future research in the current direction can be devised based on the discussion outcomes.

## Introduction

Abiotic and biotic stresses (in isolation and/or combination) are known to cause severe decline in crop productivity globally as a result of their impact on plant growth, development, and metabolism (Suzuki et al., 2014). Maintenance of plant-mineral nutrients status has been extensively evidenced to significantly improve the crop-productivity and -resistance to various stresses (Anjum and Lopez-Lauri, 2011; Gill and Tuteja, 2011). Sulfur (S) stands fourth in the list of major plant-nutrients after N, P, and K, and its importance is being increasingly emphasized in agriculture (Yi et al., 2010) and plant stress tolerance (Gill and Tuteja, 2011; Nazar et al., 2011). Nevertheless, S-deficiency in agricultural-soils is becoming widespread globally (Anjum et al., 2012a). Thus far, adopted approaches such as increased S-fertilization, -remobilization, and -uptake/accumulation may not be sufficient for S-deficiency-alleviation. Nevertheless, plant harbored-S is metabolically inert and is of no significance if it is not efficiently assimilated into physiologically/biochemically exploitable organic forms that is performed by the process of S-assimilation.

As the first committed step of primary S-assimilation in plants, ATP-sulfurylase (ATP-S; Adenylsulfurylase/ATP:sulfate adenylyltransferase; E.C. 2.7.7.4) catalyzes the activation of sulfate (SO_4_^2-^) and yields adenosine-5^′^-phosphosulfate (APS) that is reduced to sulfide (S^2-^) and incorporated into cysteine (Cys). Having thiol (S^2-^)-residue and due to its strong nucleophilic-characteristics, Cys performs important metabolic-functions and actively mediates redox-reactions (Hell and Wirtz, 2011). Notably, as a major component of predominant thiol-peptide found in plants and as a direct/indirect precursor, Cys is involved in the synthesis of S-containing compounds including glutathione (GSH, γ-glutamyl-cysteinyl-glycine) and its analog homo-GSH (h-GSH, γ-glutamyl-cysteinyl-β-Ala), reported in several genera within Fabaceae; phytochelatins (PCs; γ-glutamyl-cysteinyl)^nx^; *n* = 2-11; x represents (Gly, Ser, β-Ala, Glu, Gln, or no residue), and metallothioneins (MTs), Cys-rich gene-encoded low-molecular-weight peptides. Previous S-compounds are known for their involvement in plant-tolerance to varied abiotic–biotic stresses, and metal/metalloid-homeostasis as well (Rausch and Wachter, 2005; Verbruggen et al., 2009; Anjum et al., 2010, 2012b, 2014a,b; Na and Salt, 2011; Seth et al., 2012; Gill et al., 2013). Additionally, in secondary SO_4_^2-^-assimilation, where instead of entering the reductive S-assimilation pathway after ATP-S-mediated activation, APS is phosphorylated in a APS kinase-catalyzed reaction to produce 3^′^-phosphoadenosine 5^′^-phosphosulfate (PAPS). PAPS is involved in the production of other S-containing methionine-derived (aliphatic) or tryptophan-derived (indolic) secondary metabolites such as glucosinolates (GSs). GSs (particularly indolic type) are reported to protect plants mainly against several biotic stress-factors such as herbivory and pathogenesis, and are required for plant-immunity (Frerigmann and Gigolashvili, 2014). Therefore, S-assimilation pathway-enzymes including ATP-S are the major target of current plant-nutrition research to achieve maximum benefits including improved productivity of crops and their resistance to multiple stresses with less S-input (Herrmann et al., 2014).

Thus, to briefly highlight S-compound-mediated role of ATP-S in plant stress tolerance, ATP-S structure/chemistry and occurrence are overviewed, recent literature available on ATP-S roles, regulations and underlying major mechanisms in plant abiotic and biotic stress tolerance is appraised, ATP-S intrinsic regulation by major S-compounds is summarized, and important open-questions in the topic considered are highlighted herein.

## ATP-S: Structure/Chemistry and Occurrence

Described as monomers or homo-oligomeric complexes (which do not require GTPase for activation), plant-ATP-S has been reported to be a homotetramer of 52–54 kDa polypeptides, or a mono-functional, non-allosteric homodimer (100 kDa, formed by two ∼48 kDa monomers; Phartiyal et al., 2006; Ravilious et al., 2013; Bohrer et al., 2014; Koprivova and Kopriva, 2014; Prioretti et al., 2014). Photosynthetic organisms can exhibit a variable number of ATP-S isoforms (Koprivova and Kopriva, 2014; Prioretti et al., 2014). X-ray crystal structure of *Glycine max* ATP-S isoform 1 in complex with APS revealed the exhibition of several highly conserved substrate-binding motifs in the active site and a distinct dimerization interface compared with other ATP-S (Herrmann et al., 2014). Enzymes involved in S-assimilation are not equally expressed in all plant cell-types/ organelles. In particular, ATP-S, APS kinase, serine acetyltransferase, and O-acetylserine-(thiol)-lyase are present in both plastids and cytosol but APS reductase and sulfite reductase are localized only in plastids for catalyzing the reduction steps (Lopez-Martin et al., 2008; Bohrer et al., 2014; Koprivova and Kopriva, 2014). Occurrence of SO_4_^2-^-activation in cytosol and plastids also supports the presence of ATP-S in these locations (Koprivova and Kopriva, 2014). Seed-plants possess multiple ATP-S-isoforms. Four ATP-S genes (*ATPS1, -2, -3*, and *-4*) reported in *Arabidopsis thaliana* have N^′^-terminal extensions typical of plastid-transit-peptides, and are located on different chromosomes; however, one of them can also be cytosolic (Rotte and Leustek, 2000; Prioretti et al., 2014). Genetic-identity of cytosolic-ATP-S has been verified recently (Bohrer et al., 2015). *A. thaliana* *ATPS2* was evidenced to be dually encode plastidic and cytosolic forms, where translational-initiation at AUG^Met1^ and AUG^Met52^ or AUG^Met58^ produced *ATPS2* in plastid and cytosol, respectively (Bohrer et al., 2015). *Oryza sativa* has two ATP-S genes (*ATPS1-2*; Kopriva et al., 2007). Plastidic and/or mitochondrial localization of ATP-S genes (*Glyma10g38760*, *Glyma20g28980*, *Glyma13g06940*; *Glyma19g05020*) was reported in *G. max* (Yi et al., 2010).

## ATP-S: Roles and Regulations in Plant Abiotic Stress Tolerance

ATP-sulfurylase can be involved in plant-tolerance to several abiotic stresses *via* different S-compounds. GSH, a non-protein S-containing tripeptide acts as a storage and transport form of reduced-S. Significant induction of GSH-based defense-system, its role in reactive oxygen species (ROS)-scavenging, and in the maintenance of reduced cellular-redox environment have been extensively evidenced in plants under various abiotic stresses including metal/metalloids (Anjum et al., 2010, 2012b, 2014a,b; Gill and Tuteja, 2010; Noctor et al., 2012; Talukdar, 2012; Gill et al., 2013; Talukdar and Talukdar, 2014) and salinity (Ruiz and Blumwald, 2002; Kocsy et al., 2004; Gill and Tuteja, 2010; **Table 1**). Cys-rich metal-chelating proteins – MTs and PCs maintain homeostasis of varied metals/metalloids and mitigate major detrimental effects of their elevated concentrations (Na and Salt, 2011; Anjum et al., 2014a). h-GSH is an effective antioxidant in Fabaceae plants, where it is argued to scavenge ROS, act as PCs-precursor, and found to be involved in xenobiotic defenses *via* GSH-sulfotransferases (Frendo et al., 2013). GSs provide plant-tolerance to varied abiotic stresses including drought/salinity, metals/metalloids, and nutritional-deficiencies (Martínez-Ballesta et al., 2013).

**Table 1:** Summary of representative studies on ATP-S activity or expression modulation/regulation in abiotic and biotic stressed plants.

Plant species	Response	Reference
**Abiotic stresses**
Sulfate starvation		
*Arabidopsis thaliana*	–	Liang et al. (2010)
*A. thaliana*	+	Lappartient et al. (1999)
*Brassica napus*	+	Lappartient and Touraine (1997)
*Nicotiana tabacum* cultured cells	+	Reuveny et al. (1980)
*Zea mays*	+	Hopkins et al. (2004)
*Z. mays*	+	Schiavon et al. (2007)
**Cadmium**
*A. thaliana*	+	Harada et al. (2002), Weber et al. (2006)
*A. thaliana*	+	Bashir et al. (2013)
*B. juncea*	+	Lee and Leustek (1999)
*B. juncea*	+	Masood et al. (2012)
*B. juncea*	+	Asgher et al. (2014)
*B. juncea*	+	Heiss et al. (1999)
*B. juncea*	+	Khan et al. (2009a)
*Lepidium sativum*	+	Gill et al. (2012)
*Sedum alfredii* Hance	+	Guo et al. (2009)
*Thlaspi caerulescens*	+	van de Mortel et al. (2008)
*Triticum aestivum*	+	Khan et al. (2007)
**Salinity**
*B. juncea*	+	Nazar et al. (2011)
*B. juncea*	–	Khan et al. (2009b)
*B. napus*	+	Ruiz and Blumwald (2002)
**Light (irradiation)**
*A. thaliana*	–	Huseby et al. (2013)
*Avena sativa*, *Hordeum vulgare* and *Z. Mays*	+	Passera et al. (1989)
H_2_O_2_		
*B. napus*	–	Lappartient and Touraine (1997)
**Glutathione**
*B. napus*	–	Lappartient and Touraine (1996)
*Lemna gibba* and *Salvinia minima*	+	Leao et al. (2014)
**Chilling/Cold stress**
*Glycine max*	+	Phartiyal et al. (2006)
*Z. mays*	+	Nussbaum et al. (1988), Brunner et al. (1995)
**Biotic Stress**
Infection by *Phytopthora infestans* and/or *Botrytis cinerea*
*A. thaliana* and *B. juncea*	+	Matthewman (2010)

*+, – signs indicate increase or decrease, respectively.*

Varied abiotic stresses differentially regulate ATP-S activity/expression in plants (**Table 1**). Among metals/metalloids, literature is full on Cd-accrued enhanced ATP-S activity and increased pools of Cys and GSH (Guo et al., 2009; Khan et al., 2009a; Masood et al., 2012; Bashir et al., 2013; Asgher et al., 2014). Up-regulation of ATP-S transcripts was reported in Cd-exposed *Brassica juncea* (Heiss et al., 1999) and *A. thaliana* (Harada et al., 2002). Enhanced ATP-S activity was evidenced in several Cd/Zn-hyperaccumulators including *Sedum alfredii* (Guo et al., 2009), *A. halleri* (Weber et al., 2006), and *Thlaspi caerulescens* (van de Mortel et al., 2008). Lower ATP-S activity-exhibiting *Brassica juncea* cv. (SS2) was reported to be salt-sensitive (Khan et al., 2009b). Chilling-stress can also mediate modulation of levels and also intercellular-distribution of ATP-S mRNAs (Kopriva et al., 2001). Reports also indicate the ATP-S activity/expression-regulation by light-regimes. Forty four hours of dark was reported to down-regulate *ATPS1*–*ATPS3*; whereas, *ATPS4* was not affected (Huseby et al., 2013). However, after 3-h of re-illumination, *ATPS1*, *ATPS3*, and *ATPS4* were induced by light but only *ATPS2* reached the levels in control plants (Huseby et al., 2013).

Unknown for its essential-function in higher plants, Se, taken-up as selenate (SeO_2_^-4^)/or selenite (SeO_2_^-3^) was reported to enhance plant growth and antioxidant activity (Pilon-Smits and Quinn, 2010). ATP-S is also involved in Se-reductive-assimilation pathway and activates SeO_2_^-4^ to organic-metabolite, seleno-Cys (El Kassis et al., 2007; Pilon-Smits and Quinn, 2010). Recently, ability to hyperaccumulate and hypertolerate Se in *Stanleya pinnata* (Se-hyperaccumulator) was considered due to its potential to exhibit higher transcript levels of *APS1*, *APS2*, and *APS4* (vs. *Brassica juncea*, a non-Se-hyperaccumulator; Schiavon et al., 2015). Additionally, under Se-exposure and S-deficiency, *S. pinnata* hyperaccumulates and tolerates Se due to its ability to convert SeO_2_^-4^ to non-toxic organic-seleno-compounds by down-regulating *APS1*, *APS2*, and *APS4*. However, under S-sufficient and Se-exposure, adoption of different types of regulatory mechanisms and subcellular-localization were revealed in *S. pinnata* and *Brassica juncea*, where Se up-regulated *APS1* and *APS4* but was not able to affect *APS2* in *S. pinnata* (Schiavon et al., 2015). Earlier, compared to *Camellia sinensis* grown on Se un-enriched soil, young (or mature) leaves and roots were reported to exhibit a lower and higher *APS1* and *APS2* expression levels in Se-enriched soil-grown *C. sinensis* (Tao et al., 2012).

Extensive reports are available on S-depletion-mediated regulation of ATP-S activity/expression. ATP-S isoforms can be differentially expressed by S-depletion. *AtAPS3* increased in S-deprived *A. thaliana* (Liang et al., 2010; Kawashima et al., 2011). However, response of *AtAPS2* (a putative cytosolic-isoform) to S-depletion is inconsistent between different studies (Logan et al., 1996; Takahashi et al., 1997; Kawashima et al., 2011). Plant-ontogeny/developmental-stages can also modulate ATP-S-activity/expression under S-depleted conditions (Rotte and Leustek, 2000; Honsel et al., 2012). Confirmed by ATP-S protein-immunoblotting, ATP-S-activity exhibited a linear, threefold decline between 14 and 61 days after germination in S-depleted *A. thaliana* (Rotte and Leustek, 2000). Compared to young leaves, higher transcript-levels of *PtaATPS3/4* were reported in *Populus tremula*×* Populus alba* after 21 days of S-depletion (Honsel et al., 2012). Contrarily, S-depletion did not lead any change in *PtaATPS1/2*-expression in young leaves; whereas, this ATP-S isoform increasingly expressed after 9 days in mature leaves (Honsel et al., 2012). In *A. thaliana*, both S-deficiency (-S/-Cd) and Cd (+S/+Cd) regulated APT-S activity (Bashir et al., 2013).

ATP-S gene-regulation has been discussed in different SO_4_^2-^-starved plants. *APS1*, *APS3*, and *APS4* genes can be targeted to regulate root-shoot-SO_4_^2-^-accumulation by miR395 (small conserved non-coding RNAs with 20–24 nucleotides, specific sizes, and dedicated functions; Liang and Yu, 2010; Liang et al., 2010). In *APS4-RNAi* transgenic *A. thaliana*, loss-of-function of *APS1* or/and *APS4*-genes can lead to 5-times higher SO_4_^2-^-accumulation in shoot (vs. wild-type plants). Additionally, enhanced miR395-expression in the absence of *APS4* was considered as an indicator of a negative-feedback-loop between miR395 and *APS4* (Liang et al., 2010). Moreover, unlike *APS1* and *APS4*-mRNA, both miR395 and *APS3* can exhibit a similar response to SO_4_^2-^ starvation; however, *APS1* and *APS3*-expression can be regulated *via* miR395 (Liang and Yu, 2010). MiRNA395 can also cleave mRNAs encoding *ATPS1* and *ATPS4*-isoforms (Jones-Rhoades and Bartel, 2004; Kawashima et al., 2009). Nevertheless, *ATPS1* and *ATPS4* were evidenced as the major targets of miRNA (miR395), in both leaves and roots (Kawashima et al., 2009). In a similar report, SO_4_^2-^-limitation decreased *ATPS4*-transcript-levels; whereas, *ATPS1* levels were unaffected (Kawashima et al., 2011). It was argued in previous and other studies that for the SO_4_^2-^-limitation-mediated decreased *ATPS4*-transcripts that *ATPS4* can undergo a canonical regulation by miR395 because its mRNA levels can decrease following miR395-induction (Kawashima et al., 2009, 2011; Liang et al., 2010). SO_4_^2-^-deficiency cannot affect (Kawashima et al., 2011) or can bring a slight decrease in the levels of *ATPS1* mRNA (Liang et al., 2010). ATP-S activity/expression can also be controlled/modulated by S-Limitation 1 (SLIM1), a TF identical to Ethylene-Insensitive3-Like (EIL3) TF in *Arabidopsis* and the regulator of many S-deficiency responsive genes (Wawrzynska and Sirko, 2014). ATP-S-relation with ethylene is supported by the role of EIN3 and EIL1, two members of EI3/EIL TF family as central regulators of ethylene signaling (Maruyama-Nakashita et al., 2006). Relation among ATP-S-activity, GSH-content, ethylene-level, and decreased Cd-impacts was reported in Se-supplemented Cd-exposed *Triticum aestivum* (Khan et al., 2015). Nevertheless, a joint action of miR395 and SLIM 1 TF can maintain optimal-levels of ATP-S-transcripts in S-starved plants (Kawashima et al., 2011).

## ATP-S: Roles and Regulations in Plant Biotic Stress Tolerance

Through different S-compounds such as Cys, GSH, and GSs, ATP-S is also involved in plant-tolerance to several biotic stresses. Free-Cys and cytosolic Cys-homeostasis can orchestrate plant-pathogen responses (Gullner and Kömives, 2001; Álvarez et al., 2012). Pathogen-infection can trigger accumulation of GSH and also the modulation of transient changes in its redox-state (Noctor et al., 2012). Elevated GSH and Cys were reported to suppress and delay virus-symptoms, and decrease virus-content in zucchini yellow mosaic virus (ZYMV)-infected *Cucurbita pepo* (Zechmann et al., 2005, 2007; Zechmann and Müller, 2008; Király et al., 2012). Decreased GSH-pool and its redox-state in *Lycopersicon esculentum* signify their role against *Botrytis cinerea* infection (Kuźniak and Skłodowska, 2005). Elevated GSH metabolism can also counteract infection in plants with tobacco mosaic virus (Höller et al., 2010; Király et al., 2012), *Pseudomonas syringae* (Großkinsky et al., 2012) and *B. cinerea* (Simon et al., 2013). Zechmann (2014) recently reviewed the compartment-specific importance of GSH in biotic stressed plants.

Evidences confirm the requirement of a certain level of GSH for disease-resistance *via* synthesis of pathogen defense-related molecules such as camalexin, an indole-phytoalexin containing one S-atom per molecule with partly Cys-derived thiazole-ring (Noctor et al., 2012). A link between GSH-deficiency and plant-susceptibility to pathogens such as *Pieris brassicae* was reported in *A. thaliana phytoalexin deficient 2-1 (pad2-1)* mutant (Dubreuil-Maurizi and Poinssot, 2012). Earlier, a higher susceptibility of previous GSH-deficient-mutant to insect-herbivore *Spodoptera littoralis* was related with a lower GSs-accumulation therein (Schlaeppi et al., 2008). GSH (and also numerous GSH-sulfotransferases) is required for wound-induced resistance to *B. cinerea* (Chassot et al., 2008; Consonni et al., 2010). Expression of defense-related genes including *PATHOGENESIS-RELATED1* (*PR1*) can be activated by exogenous-GSH-mediated mimicking of fungal-elicitors (reviewed by Noctor et al., 2012). Inner chloroplast-envelope-transporters export GSH across the chloroplast envelope. In *Arabidopsis*, *CLT1*, *CLT2*, and *CLT3* genes encode these transporters (Maughan et al., 2010). Decreased *PR1*-expression and also lower resistance to the oomycete *Pieris brassicae* were reported in CLTs-defective *Arabidopsis*-mutants (Maughan et al., 2010). Numerous reports support an increased S-requirement in plants infected with *Phytopthora infestans* and/or *B. cinerea* and was advocated to be met, at least in part, by increased transcription of *ATPS1*, *ATPS3*, and *ATPS4* genes (Matthewman, 2010). To this end, in *A. thaliana*, ATP-S genes namely *ATPS1* and *ATPS3* were reported to be linked with the regulation of biosynthetic networks of aliphatic and indolic GSs, respectively (Yatusevich et al., 2010). *P. infestans* and *B. cinerea*-infection in *A. thaliana* resulted in a similar increase in the transcript levels of *ATPS1*, *ATPS3* and *ATPS4* (Matthewman, 2010). Earlier, *B. cinerea*, *P. Infestans*, and aphid *Myzus persicae* were reported to induce a GSs-response in *Arabidopsis* (Kim and Jander, 2007; Rowe et al., 2010).

## ATP-S: Intrinsic Regulations by S-Compounds

**FIGURE 1:**
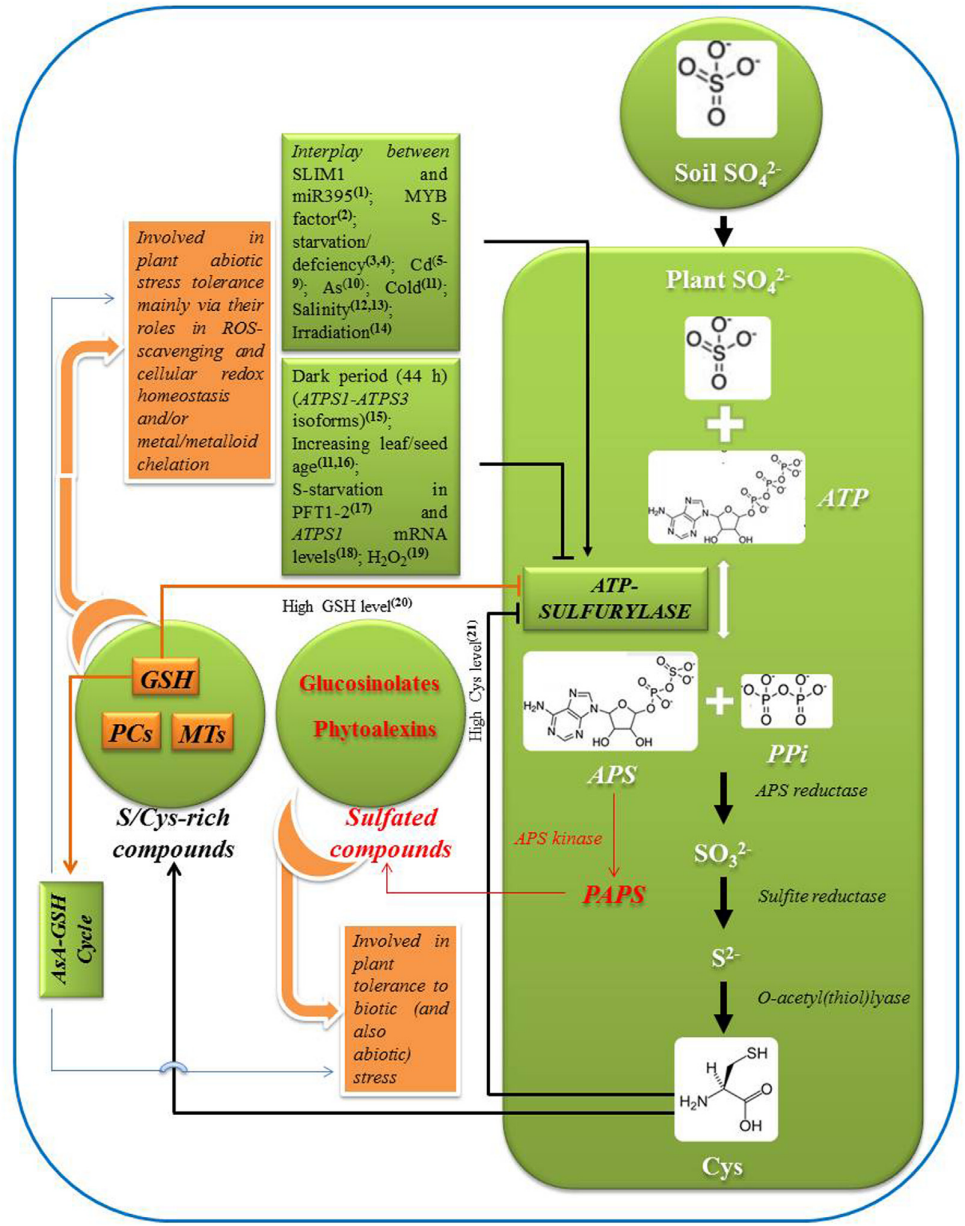
**Schematic representation of pathway of sulfate assimilation, reaction catalyzed by ATP-sulfurylase (ATP-S), and its regulation by major factors.** Role of ATP-S in plant stress tolerance through sulfur/cysteine rich and sulfated compounds is outlined. Positive and negative regulation of ATP-S is indicated by arrows and blunt ends, respectively, [^1^Kawashima et al. (2011); ^2^Yatusevich et al. (2010); ^3^Hopkins et al. (2004); ^4^Schiavon et al. (2007); ^5^van de Mortel et al. (2008); ^6^Guo et al. (2009); ^7^Gill et al. (2012); ^8^Bashir et al. (2013); ^9^Asgher et al. (2014); ^10^Leao et al. (2014); ^11^Phartiyal et al. (2006); ^12^Ruiz and Blumwald (2002); ^13^Nazar et al. (2011); ^14^Passera et al. (1989); ^15^Huseby et al. (2013); ^16^Rotte and Leustek (2000); ^17^Takahashi et al. (1997); ^18^Liang et al. (2010); ^19^Lappartient and Touraine (1997); ^20^Lappartient and Touraine (1996); ^21^Vauclare et al. (2002)]. (APS, adenosine 5^′^-phosphosulfate; Cys, cysteine; AsA, ascorbate; GSH, reduced glutathione; PCs, phytochelatins; MTs, metallothioneins; ROS, reactive oxygen species).

Literature is scarce on insights into S-compounds-mediated regulation of ATP-S activity/expression in plants. Among the thiol-compounds, GSH, rather than Cys can be used as a signal for regulating ATP-S (Lappartient et al., 1999; Vauclare et al., 2002). Externally supplied GSH-mediated increase in Cys and GSH accumulation can control both ATP-S activity and SO_4_^2-^-uptake (Vauclare et al., 2002). Compared to its lower level (up to 1.0 mM), Cys can significantly decrease ATP-S-activity at its higher level (2.0 mM). However, further increase in Cys-concentration can cause an additional accumulation of GSH that in turn can cause a decrease in ATP-S-mRNA, -protein, and -activity (Lappartient et al., 1999; Vauclare et al., 2002). ATP-S enzymatic activity might be sensitive to redox regulation in plants, where it can be a target for thioredoxins (reviewed by Prioretti et al., 2014). As a major redox regulator, GSH feeds into glutaredoxin system and subsequently into the thiol-redox-network (Dietz, 2008). Referring to the studies of Lappartient and Touraine (1996, 1997), cellular-redox-conditions and also that of GSH were advocated to modulate ATP-S-activity (reviewed by Yi et al., 2010). However, the authors suggested further biochemical- and structural-analysis of ATP-S to determine how, and to what extent, ATP-S responds to redox-changes. MiR395 is related with ATP-S-genes such as *APS1*, *APS3*, and *APS4* (Liang et al., 2010). Recently, GSH-supplementation was reported to block accumulation of S-deprivation-inducible miR395 in S-deprived *A. thaliana* (Jagadeeswaran et al., 2014). Declined GSH-pools and induced miR395-levels in S-deprived *A. thaliana* were cross-talked (Kawashima et al., 2011; Matthewman et al., 2012). Nevertheless, biosynthesis of indolic-GSs in *A. thaliana* is regulated by MYB34, MYB51, and MYB122 TFs (Frerigmann and Gigolashvili, 2014). In *A. thaliana*, expression of both *ATPS1* and *ATPS3* isoforms was reported to be controlled by all six GSs-related MYB TFs namely MYB28, MYB29, and MYB76; MYB51, MYB34, and MYB122 (Yatusevich et al., 2010). *ATPS1* and *ATPS3* were expected to be strongly associated with the control of synthesis of aliphatic and indolic GSs, respectively. *A. thaliana* overexpressing or disruption in MYB51-gene showed alterations in ATP-S-transcript-levels and -activity (Matthewman, 2010; **Figure 1**).

## Conclusion and Open Questions

S-containing compounds such as Met, GSH, h-GSH, PCs, and GSs, directly or indirectly modulated/regulated by ATP-S are involved in plant tolerance to both biotic and abiotic stresses. Much has been achieved on the subject considered herein; there remain numerous aspects to be enlightened and open-questions to be answered. Ample scope exists for getting more molecular-genetic insights into the energetically unfavorable-reaction that yields APS from SO_4_^2-^ and ATP with ATP-S-catalytic-function. Notably, compared to APR enzyme and its encoding genes, much less amplitude and significance has been given to ATP-S in mutant-experiments. Hence, molecular-genetic dissection of so far neglected significance of ATP-S as a major control in the initial step of S-assimilation pathway is required. ATPS has been evidenced as an integral part of GS-biosynthesis-regulatory network (Matthewman, 2010); however, unveiling insights into interrelationship of ATP-S transcripts with other secondary S-assimilation products will be rewarding. Though picture is clear regarding the relationship of *ATPS1* and *ATPS3*-expression with MYB TFs (Yatusevich et al., 2010) effort is required to unveil potential relationships of MYB TFs with *ATPS2* and *ATPS4*-expression (Prioretti et al., 2014). If done, these studies may shed light on the complexity of regulatory interactions between primary and secondary S-metabolism. Efforts are also required to dissect the molecular biology/genetics of interaction of ATP-S with ratios of oxidized and reduced GSH (GSSG/GSH) and that of oxidized (dehydroascorbate, DHA) and reduced ascorbate (AsA; DHA/AsA) in stressed plants since DHA can be recycled back to AsA at the expense of GSH (or NADPH) by the AsA–GSH cycle-enzymes (Anjum et al., 2010). Role of miR395 family of micro-RNAs in the regulation of *ATPS1-4* is known (Maruyama-Nakashita et al., 2006; Kawashima et al., 2011); however, picture is unclear in context with functions and effects of miR395 on *ATPS3* and *ATPS4*-expression. A cross-talk among GSH-pools, miR395-levels and ATP-S-transcripts/activity particularly under deprived condition of interdependent nutrients S and N can also be significant for the maintenance of the status of S-compounds, and S-N homeostasis.

## Acknowledgments

NA is grateful to the Portuguese Foundation for Science and Technology (FCT) financial support in the form of post-doctoral research grants (SFRH/BPD/64690/2009; SFRH/BPD/84671/2012). SG and RG acknowledge the funds from DST-SERB, CSIR, and UGC, Government of India, New Delhi. Authors apologize if some references related to the main theme of the current article could not be cited due to space constraint.
